# Coupling coordination analysis of low-carbon development, technology innovation, and new urbanization: Data from 30 provinces and cities in China

**DOI:** 10.3389/fpubh.2022.1047691

**Published:** 2022-11-15

**Authors:** Ying He, Guolei Liu

**Affiliations:** Faculty of Political Science, School of Public Administration, Central China Normal University, Wuhan, China

**Keywords:** low-carbon development, technology innovation, new urbanization, coupling coordination degree, spatial autocorrelation

## Abstract

Technology innovation capability as an endogenous driving force plays an increasingly important role in the low-carbon transformation of new urbanization. This paper's purpose is to delve into the coupling coordination relationship among the three variables, and promote system's and region's synergy development. Based on the coupling coordination degree model, spatial autocorrelation model and obstacle degree model, this paper investigated the coupling coordination of low-carbon development (LCD) quality, technology innovation (TI) capability and new urbanization (NU) level in China from 2009 to 2019. The results indicate: (1) The coupling coordination degree (CCD) of LCD quality, TI capability and NU level in all regions of the country were fluctuating for a long time, and the regions that reach the coordinated development level showed a slow rising trend with obvious regional differences. (2) Three subsystems' CCD showed significant spatial correlation characteristics, and the degree of spatial agglomeration was constantly increasing. (3) The obstacles affecting the systems' synergy mainly reflected in economic and social indexes. In the end, this paper proposed that policy coordination and linkage should be strengthened, emphasizing the integrated development of the three subsystems. It is necessary to formulate development plans in combination with geographic location and resource endowment to enhance the regional driving effect.

## Introduction

With the acceleration of industrialization in recent years, the greenhouse effect has increased, which has caused a series of serious problems such as global warming and frequent extreme weather. In the context of global climate change, the low-carbon development model is considered the best choice for all countries in the world (1). According to statistics, since the mid-twentieth century, about 90% of greenhouse gas emissions have been attributed to human activities and behaviors in urban areas (2). Cities play an important role in LCD (3). As a result, countries around the world have taken measures to address climate change and ecological breakdowns, advocating the reduction of greenhouse gas emissions and shifting to low-carbon cities for sustainable urban development (4, 5). Low-carbon development (LCD) and urbanization have been becoming important issues in the field of climate change (6).

China is the world's largest developing country, energy consumer and carbon dioxide emitter (7). The construction of new urbanization (NU) through low-carbon means has gradually become the main way of urbanization development in China. Unlike European and American countries, China's urbanization starts late. It faces more stringent environmental protection requirements and low-carbon emission requirements. China's NU requires both urbanism and carbon emission control. Also, it requires modernization while preserving heritage culture. All these have posed great challenges to China's LCD and NU construction. China has experienced a rapid urbanization over the past three decades (6). In addition to the dramatic increase in carbon emissions brought about by increasing urban population density, accelerating production practices and surging infrastructure needs, the over-reliance on energy resource inputs and production scale expansion for industrial development have also led to serious damage to the ecological environment (8). However, with the rapid development of modern information technology, the integration of scientific technology with low carbon has gradually entered people's research horizon. LCD technologies is not only necessary for mitigating and adapting to climate change, but also for ensuring the security of energy supply and building a resource-saving and environment-friendly society. Meanwhile, the central government is paying more and more attention to technology innovation (TI) with the socio-economic development. Regional TI is an important technological guarantee for regional sustainable development. Improving the regional TI capability is an important way to enhance the ability of regional health and sustainable development (9). Therefore, the TI capability is also playing an increasingly important role in NU construction and LCD.

In the context of carbon neutral and peak carbon dioxide emissions, the only way to achieve this goal is scientific and technological support, which relies on major technology innovation breakthroughs. However, due to the differences in regional development, there is a big gap between regional NU level, LCD quality and TI capability. On the basis of NU, the combination of LCD and TI will effectively increase the supply of low-carbon products and green services. At the same time, it will also guide the green consumption and industrial transformation to realize the regional and even national socio-economic green transformation and modernization development. Obviously, what needs to be clarified at this time is whether there is a link between TI capability, NU level and LCD quality in China's development process over the years? To what extent do they interact and influence each other? Is there a spatial correlation? The existing studies have not been clearly elaborated. Therefore, this paper's purpose is to explore the coupling coordination relationship and development characteristics among TI capability, LCD quality and NU level in various provincial administrative regions in China, and to explore regional differences and optimization strategies of low-carbon city construction and low-carbon technology development.

As the second largest economy in the world, China's green transformation plays a very important role in the global green transformation. Moreover, the great achievements of NU with Chinese characteristics and the concept of people-oriented urbanization construction have become a benchmark of global significance. China's LCD and NU both focus on low-carbon economy and social transformation, advocate clean energy and low-carbon lifestyles, which aims to realize the coordinated development of industrialization and ecological environment (10). China's advanced experience in coordinating the relationship between ecological civilization, technological progress and socio-economic development, and leading the green transformation and urbanization development with scientific and technological innovation will bring great reference significance to the world, especially developing countries. The data from 2009 to 2019 are mainly obtained from *China Statistical Yearbook, China Education Statistical Yearbook, China Science and Technology Statistical Yearbook, China Energy Statistical Yearbook, China Environmental Statistical Yearbook, China Population and Employment Statistical Yearbook, China Health Statistical Yearbook* and *China High Technology Industry Statistical Yearbook*. Due to the absence of some data in Tibet, Taiwan, Hong Kong and Macao, they are not included in the statistical scope.

This article proceeds as follows: In the second section, we introduce the literature review of the three subsystems of LCD quality, NU level and TI capability, and then clarify the research contents of the existing studies. The third part introduces the construction of the index system and models. The fourth part is an empirical study. It firstly compares and analyzes the comprehensive development level of each subsystem. Secondly, the evolution of CCD in each region is analyzed from the view of space-time. Its spatial correlation is verified by combining global autocorrelation and local autocorrelation. Thirdly, this paper clarifies the indexes affecting the synergistic development of each system based on the obstacle degree model. Section 5 discusses the results and proposes policy recommendations. Meanwhile, the theoretical and practical implications, limitations and future research directions of this study are elaborated.

## Literature review

### Low-carbon development

The UK government put forward the concept of “*low carbon*” in its *Energy White Paper: Our Energy Future—Creating a Low Carbon Economy* in 2003 (11). In the book, the new concept of “*Low-Carbon Economy*” explained the challenge of global warming to human survival. How to promote the “*Green Economy*” has become an urgent task. Subsequently, low-carbon concept gradually received widespread attention from governments and academia. In 2007, Japanese government advocated the concept of “*No Low-carbon Society, No Low-carbon Economy*” and introduced the concept of “*Low-Carbon Society*” (5). On June 5, 2008, the United Nations Environment Program (UNEP) proposed a “*Low-Carbon Lifestyle*” for individuals. In 2010, Chinese scholars were the first to propose the concept of “*Low-Carbon Cities*” in mainland China based on the above-mentioned concepts of western developed countries. In 2021, China launched the research report “*Carbon Neutral: China in Action*” to proactively explore China's low-carbon transformation and upgrading path. Currently, research on low-carbon is mainly focused on “*Low-Carbon City*,” “*Low-Carbon Life*,” “*Low-Carbon Technology*,” and “*Energy Consumption and Regeneration*”, etc. (12–14). In recent years, research has extended to the dimensions of “*Green Building*” (15), “*Green Logistics*” (16), “*Low-Carbon Competitiveness*” (17), and “*Carbon Emission Reduction*”. For countries around the world, LCD is a sustainable development model characterized by low energy consumption, low pollution and low emissions, which plays an important positive role in economic and social progress (3). However, for a country, cities are the basic unit of economic development. As the engine of future economic growth, cities play an increasingly important role in LCD (18). Therefore, low-carbon city construction is an important way to achieve sustainable development strategies and promote social wellbeing (5).

How should the regional LCD quality be measured? Generally speaking, if a region achieves economic growth with lower carbon emissions per unit of GDP, the region will have the higher LCD quality. In today's world, LCD takes various forms. London focuses on energy efficiency projects. Copenhagen and the United States focus on promoting the production and application of renewable energy. Germany strongly supports environmental protection industries. Japan advocates the development of low-carbon innovative technologies. While, China encourages investment in clean energy. It implies that the LCD concept involves not only greenhouse gas emissions, energy consumption and regeneration, but also economic, environmental and social production (18). On this basis, the evaluation index system of LCD quality should also be a unified subject involving the coordinated and orderly development of many aspects (18, 19). As a result, a large number of scholars have extensively explored its evaluation system in terms of economy, society, environment, resources, urban design and people's quality of life. Jia et al. (11) established the evaluation system of LCD quality from carbon emission status, carbon source's control level, carbon capture capacity, human development index and urbanization level. Tan et al. (18) established an evaluation index framework for low-carbon cities from the perspectives of economy, energy pattern, society and life, carbon and environment, urban transportation, solid waste and water.

### Technology innovation capability

The coordinated promotion of innovation and economic development is an inevitable issue for Chinese cities (20). Innovations arise from complex interactions between actors with complementary capabilities (technical competence, leadership, or investment level). These actors include businesses, research organizations, government departments, non-governmental organizations, and other intermediary organizations. They contribute to the development and diffusion of innovation (21). Generally speaking, technological talents mainly refer to enterprises, schools, research institutes, and some natural persons with invention patents and innovative technologies. They have the comprehensive strength of invention and innovation in a certain scientific and technological field. Universities, research institutions and industries are important subjects of TI (22). Knowledge structure, R&D experience, technological equipment and innovation spirit of scientific researchers will have an impact on the organization's TI capability. Technological change is the main source of productivity growth. R&D investment and R&D activities play a key role in TI progress (23). Improving the TI capability will directly enhance local industrialization, attract foreign investment and promote employment. There is a positive effect of TI capability on enterprises competitiveness, especially green technology innovation capability (24). Xie et al. (25) used dynamic panel generalized moment estimation to find that technology introduction had a negative effect on the green transformation of industries in resource-based cities, while TI could have a positive effect. In the short term, TI promotes sustainable regional development, while in the long term, the two remain in dynamic equilibrium.

It is evident that TI capability is a powerful driving force leading the cities' development (26). The evaluation system of TI capability has been expanded with the depth of research, and many research institutions have assessed it in terms of technological innovation input, output, and benefit, etc. Chen et al. (27) constructed a system of urban TI in terms of regional economic growth, research expenditure, manpower, R&D personnel's quality, number of patents granted and transportation facilities levels. Yang et al. (28) integrated economic, environmental and social benefits on this basis, and then studied the coupling coordination relationship between TI system and ecological environment system.

### New urbanization level

Urbanization is a process of urban spatial expansion in which non-agricultural industries and non-agricultural populations cluster in towns and cities (29). The proportion of the population living in urban areas is a key index of modernization. It is used to represent the urbanization level in this area. Urbanization is more closely related to the level of productivity, industrialization and technology of a region. Urbanization at the conceptual level includes demographic urbanization, economic urbanization, geospatial urbanization and social civilization urbanization, involving industrial economy, human geography, urban construction and other aspects. Compared with the traditional urbanization concept of “*Object-Oriented*”, the NU insists on the “*People-Oriented*” concept and emphasizes that all people should enjoy the fruits of urbanization together. China's NU not only requires the harmonization of population, land and economy, but also advocates the protection of ecological environment and LCD of the region (30, 31).

Scholars' studies on the coupling coordination of NU mainly focus on the coupling relationship between carbon emissions, land use efficiency and ecological environment system (32). Tian et al. (33) analyzed the spatial and temporal coupling interactions between urbanization and ecosystem services in the Beijing-Tianjin-Hebei region using a CCD model and a spatial autocorrelation statistical model. The region was encouraged to strengthen sustainable ecological network management and mutually beneficial cooperation across administrative boundaries, and subsequently promote sustainable regional development. On this basis, Gan et al. (34) studied the coupling coordination of cities, population and industries as a way to explore the level of urbanization-industry integration, and found that the low-level and slow-development of industrial subsystems would lead to a poorly coordinated level of urbanization-industry integration. However, a city's radiation capacity has a great impact on the industrial structure and product distribution of the surrounding areas (29), so the study emphasizes the radiation role of central cities to promote the region's synergetic development (34). Therefore, the NU construction should pay more attention to the coordinated development of economy, society and environment. It is important to reduce energy consumption and improve energy productivity.

Scholars have found that environmental pressure is higher in cities (35, 36). In the coupling relationship between urbanization and environmental systems, social urbanization and environmental regulation are key factors in decision making to adjust coordinated development (37). Zhao et al. (35) affirmed the important role of ecological levels when studying the relationship between cities and the environment based on data collected from 209 countries and regions worldwide. It also pointed out that economic urbanization had a positive impact on the coupling of the two systems, with higher income levels tending to exhibit a more harmonious and coordinated relations. It is thus clear that the changes in productive activities of urban populations brought about by urbanization have an important impact on the structure and function of urban ecosystems (29). However, in the future, the majority of the world's future population will live in urban areas, and cities will gradually become the focus of sustainable development in all countries (38). In this context, NU has become a necessary path to modernization for all countries and a strong driving force for regional economic development. As a result, more and more scholars have started to inject the evaluation elements of “*people-oriented*” and “*green-coordinated development*” on the basis of traditional urbanization research (34, 39–41). For example, Wu and Zhao (41) selected 26 indexes from four aspects: economic, social, environmental, and urban-rural coordination to comprehensively measure the NU level of each city. Gan et al. (34) assessed it in terms of transportation level, public services, environmental management, population employment, living standard, income expenditure level and industrial scale.

### Variable linkage and research methods backtracking

With the deepening of the connection between NU and LCD, some scholars hold that urbanization promotes carbon emissions (42). The reason is that population growth during rapid urbanization will bring increased demand for transportation and infrastructure. China's energy consumption will also expand at an unprecedented rate, accompanied by high coal-based energy consumption and high carbon dioxide emissions (43, 44). For instance, Zhu and Peng (14) found that consumption level was highly correlated with carbon emissions, and population urbanization was the key factor for the increase of carbon emissions in China. Increasing population, wealth and urbanization level would increase carbon dioxide emissions (10). With the continuous acceleration of urbanization, cities have a growing number of people and productive assets. In addition, the increase in household consumption and the expansion of urban infrastructure would lead to rapid urbanization, which will most likely result in more carbon emissions (6). Some other scholars consider that the increase in urbanization rate inhibits carbon emissions and has a negative effect on the carbon emissions efficiency. Meanwhile, energy intensity and carbon intensity decrease with the development of urbanization and the advancement of industrial structure (43). Urbanization is accompanied by rapid economic development. China's economy will grow faster than energy consumption and carbon dioxide emissions for a certain period of time (43). Subsequently, the coordination level and spatial correlation between urbanization and the ecological environment will be constantly strengthened, and the balance between the two will reach beyond expectation (45, 46). Many provinces are committed to raising the LCD level and accelerating the construction of low-carbon cities. Low-carbon cities are characterized by economies of scale and regional differences. The larger scale of urbanization, the more perfect infrastructure and the higher level of technology will lead to the greater positive effect of green growth in the region (47).

For regional development, however, technological empowerment will be even more influential. It will effectively spawn a new batch of smart cities and smart governments. Smart cities imply the use of science and technology and the collective intelligence of cities to achieve smarter urban mobility, more robust infrastructure, and more interactive urban management systems (48). For example, cities can use new industrial and communication technologies to develop energy-efficient buildings and smart energy grids (49, 50). Smart cities involve technological, social, and political processes aimed at solving public problems using more advanced technologies, providing innovative urban services, improving the quality of life of citizens, and achieving healthy and sustainable urban development. Moreover, urban innovation is not only dependent on local innovation activities, but is also enhanced when cities are deeply integrated into intercity innovation networks (51). Moreover, the TI capability would offset the negative impact of urbanization on the environment and reduce carbon emissions (61). The TI capability not only provides practical methods for realizing low-carbon and zero-carbon cities from the technical level, but also plays an important role in promoting the development of green and low-carbon industrialization. It can be seen that the three variables are mutually promoting and interacting in theory and practice. In the construction of NU, LCD and TI provide a new focus and impetus for urban construction and national development from the perspective of concept strengthening and technological empowerment.

Among the existing research methods, the coupling studies is mainly based on coupling coordination model and gray multivariable coupling model, supplemented by relative development degree model and spatial autocorrelation model. Exploring the relationship between variables depends on empirical studies and case studies. The advantages and limitations of different research methods are shown in Table 1. Combined with the research purposes, we choose to combine the coupling coordination degree model and spatial autocorrelation model to analyze the coupling coordination of variables at the spatio-temporal level, and use the obstacle degree model to assist in identifying the specific indicators affecting the coupling coordination degree.

**Table 1:** Review of research methods.

**Category**	**Research methods**	**Advantage**	**Limitation**	**Related literature**
Coupling relationship between variables	Coupling coordination degree model	Analyze coordinated development level and dynamic correlations in different regions.	Unable to specify the micro indexes affecting the coordinated level.	(37, 52, 53)
	Relative development degree model	Identify subsystems that are lagging behind in the synergistic development of multiple subsystems.	Unable to clarify the magnitude of the system's CCD.	(54)
	Multivariable coupling model	Eliminate overlapping information; Calculate coordination coefficients; Reflect the positive/negative interaction between the indicators.	Mainly revolves around independent systems; Less used in regional comparisons.	(55)
	Cointegration model	Clarify the long-run equilibrium relationships between different systems/factors; Clarify the magnitude of the impact.	Difficult to reflect regional differences; Unable to estimate the coupling coordination size.	(56)
	Spatial autocorrelation model	Spatial dependency judgment; Measures the degree of aggregation and dispersion of spatial element attributes.	Mainly used as a complementary analysis to explore the spatial correlation characteristics of coupling coordination.	(31, 46)
Influence mechanism between variables	Literature review; Empirical studies	Explore the mechanism of influence between independent variables; Identify mediating and moderating variables.	Unable to verify the association effects of variables in times and space.	(57, 58)

### Literature summary and research design

From the existing studies, the research on LCD has been increasingly linked to carbon emissions, energy restructuring and rural development in recent years. Admittedly, a comprehensive understanding of low-carbon emissions is the first step of LCD. Low-carbon concept has gradually penetrated into all areas of urban development, including production methods, consumption patterns, social culture and development policies (3). This requires a balance between industrialization, urbanization and low-carbonization. Therefore, an independent and complete industrial system should be established on the basis of economic development to realize the sustainable development of population, resources and environment. This coincides with the concept of NU. The current coupling research related to NU and carbon emission is gradually gaining attention, which provides guidance for the government to formulate carbon emission reduction strategies. It also provides valuable references for decision making in other regions to accelerate the construction of LCD and NU.

However, there are existing the following shortcomings: (1) Carbon emission intensity is only one dimension of LCD quality, and cannot reflect the comprehensive LCD quality of a certain region. (2) Most of the research focus on the micro-level, selecting certain provinces or large city clusters to start the research. Few studies are conducted at the national level as a whole. (3) The impact factors of TI are not included in the scope of the coupling study. (4) The research methods are relatively homogeneous. The influence factors of the coupling coordination situation have not been clarified. In view of this, what is the relationship between the level of LCD and NU? Is there a synergistic relationship between TI capability in the NU level and LCD quality of the region? What are the evolutionary paths of the coupling coordinated development levels among the three systems in time and space? Does the level of coupling coordination among the three systems have spatial correlation? What are the indexes that mainly affect the level of coordination among the systems? The above are exactly the questions that this study tries to address.

## Methodology and data

### Index system construction and measurement

In order to comprehensively reflect the overall situation of LCD quality, TI capability and NU level of China's provincial administrative region, this paper constructs the evaluation index system of these three variables (Table 2).

**Table 2:** The index system of LCD quality/TI capability/NU level.

**Target layer**	**Factor layer**	**Index layer**	**Unit**	**Effect**	**Code**
Low-carbon development quality	Economy	Gross regional product	100 million yuan	+	L1
		Secondary industry/GDP	%	–	L2
		Tertiary industry/GDP	%	+	L3
	Society	Environmental protection expenditure/GDP	%	+	L4
		Per capita daily consumption of tap water for residential use	Liter/day	–	L5
		Public buses and trolley buses per 10,000 population	Unit	+	L6
	Energy	Electricity consumption by region	100 million kW·h	–	L7
	Environment	Per capita public green areas	sq.m	+	L8
		Forest coverage rate	%	+	L9
		Daily disposal capacity of city sewage	10,000 cu.m	+	L10
		Volume of sulfur dioxide emission	10,000 tons	–	L11
		COD discharged of industry waste water	Ton	–	L12
Technology innovation capability	TI agglomeration	The R&D expenditure input intensity	%	+	T1
		Full-time equivalent of R&D personnel by region	Man-year	+	T2
		The number of R&D institutions	Unit	+	T3
	TI transformation	Scientific papers issued of higher education institutions	Piece	+	T4
		Patent in force of higher education institutions	Piece	+	T5
		Domestic patent granted by region	Piece	+	T6
	TI support	Expenditure on new products development	10,000 yuan	+	T7
		Intramural expenditure on R&D by region	10,000 yuan	+	T8
New urbanization level	Demographic urbanization	Proportion of urban population	%	+	U1
		Population density of urban area	Persons/sq.km	+	U2
		Registered unemployment rate in urban area	%	–	U3
	Economic urbanization	Per capita GDP	Yuan	+	U4
		Per capita disposable income in urban area	Yuan	+	U5
	Social urbanization	The proportion of expenditure on social security and employment in total government expenditure	%	+	U6
		Health technical personnel in health care institutions per 1,000 persons	Person	+	U7
		Public buses and trolley buses per 10,000 population	Unit	+	U8
		Per capita area of paved roads	sq.m	+	U9
	Green urbanization	Particulate matter emission in waste gas	10,000 tons	–	U10
		Amount of pollutants discharged in waste water	10,000 tons	–	U11
		Green covered area rate of completed area	%	+	U12

The LCD quality needs to consider many indexes such as economic development, social progress, living consumption, energy structure and environmental situation of the region. Low-carbon city concepts encourage the region to change to a new development model with less energy consumption, less carbon emission and high social and economic benefits (59, 60). In this paper, the LCD quality is evaluated as a subsystem from four dimensions: economic, social, energy and green environment, in which the *Secondary Industry/GDP, Per Capita Daily Consumption of Tap Water for Residential Use, Electricity Consumption by Region, Volume of Sulfur Dioxide Emission* and *COD Discharged of Industry Waste Water* are negative indexes, and all other indexes are positive indexes.

TI capability refers to the extent to which a region's government provides services to innovation subjects. It includes the specific activities and output of local industries, enterprises, higher education institutions and research institutes for TI and technology development. In this paper, we divide regional TI capability into three dimensions: TI agglomeration, TI transformation results and TI support, which are measured in terms of TI inputs, outputs and performance. All indexes are positive indexes.

This paper emphasizes that NU should focus more on ecological environment and social progress. Therefore, this paper will measure the NU level of the region in four dimensions: demographic, economy, society and ecology. The *Registered Unemployment Rate in Urban Area, Particulate Matter Emission in Waste Gas* and *Amount of Pollutants Discharged in Waste Water* are negative indexes, while all other indexes are positive indexes.

### Data processing and index assignment

In order to minimize the influence of subjective factors on the evaluation results, this paper uses the entropy weight method to determine the index weights.

Firstly, standardize the data. The *i* denotes the sample (*i* = 1, 2, …, n), and *j* denotes the index (*j* = 1, 2, …, m). *X*_*ij*_ is the original value of the *j-th* index in the *i-th* sample of a year, ${X{}^{ij}}_{\prime }$ is the standardized value, *maxX*_*ij*_ and *minX*_*ij*_ are the maximum and minimum values of the index in all spatial units.


(1) $$\begin{array}{lll} \text{Positive indexes}:\;{X_{ij}}^{\prime } & = & \frac{\;{\text{X}}_{\text{ij}}\;-\text{ min}{\text{X}}_{\text{ij}}}{\text{max}{\text{x}}_{\text{ij}}\;-\text{min}{\text{x}}_{\text{ij}}} \end{array}$$

(2) $$\begin{array}{lll} \text{Negative indexes}:\;{X_{ij}}^{\prime } & = & \frac{\text{ max}{\text{X}}_{\text{ij}}\;-\;{\text{X}}_{\text{ij}}}{\text{max}{\text{X}}_{\text{ij}}\;-\text{min}{\text{X}}_{\text{ij}}} \end{array}$$

Secondly, calculate the proportion of the *i-th* sample in the *j-th* index.


(3) $$\begin{array}{l} \rho_{ij}=\frac{X_{ij}^{{}^{\prime }}}{\overset{n}{\underset{i=1}{\sum }}X_{ij}^{{}^{\prime }}} \end{array}$$

Thirdly, calculate the information entropy of the index *e*_*j*_. N denotes the number of samples. In this paper, N = 30. To avoid lnρ_*ij*_ is meaningless, it is stipulated that if ρ_*ij*_ = 0, then $\underset{\rho_{ij}\to 0}{\lim}\rho_{ij}\ln\rho_{ij}=\;0$.


(4) $$\begin{array}{l} e_{j}=-\frac{1}{\ln\;N}\overset{n}{\underset{i=1}{\sum }}\left(\rho_{ij}\times \ln\rho_{ij}\right),\left(0\leq e_{j}\leq 1\right) \end{array}$$

Fourthly, calculate the difference coefficient *d*_*j*_ and index weight *w*_*j*_.


(5) $$\begin{array}{lll} d_{j} & = & 1-e_{j} \end{array}$$

(6) $$\begin{array}{lll} w_{j} & = & \frac{d_{j}}{\sum_{j=1}^{m}d_{j}} \end{array}$$

### Coupling coordination model

The coupling coordination model involves the calculation of three index values: the coupling degree (*C*-value), the comprehensive coordination index (*T*-value) and the coupling coordination degree (*D*-value). Finally, the system's coupling coordination status is obtained by combining the *D*-value and the coordination level division standard. The *C*-value mainly reflects the strength of correlation between systems/elements. It does not distinguish the pros and cons, nor the level of system coordination. The *T*-value is used to measure the contribution value of the overall development level of the system/factor to the coupling degree. While, the *D*-value can indicate the size of benign coupling degree in the interaction, reflecting the degree of good or bad coordination status. This study uses the *D*-value to reflect the coupling coordination degree (CCD) of interaction between the LCD quality, TI capability and NU level.

#### Comprehensive evaluation functions

Calculate the comprehensive development levels of each subsystem in different years according to the linear weighting method (Formula 7).


(7) $$\begin{array}{l} Q=\overset{n,m}{\underset{i=1,j=1}{\sum }}w_{ij}\times X_{ij}^{+}\left(or\;X_{ij}^{-}\right) \end{array}$$

#### Coupling coordination degree model

The established studies involve binary system coupling and ternary system coupling, and this study refers to the studies of Wang et al. (61), Cheng et al. (47), and Zhang and Li (62) to derive specific measurement models as follows:


(8) $$\begin{array}{l} C=\sqrt[{3}]{\frac{Q1\;\times \;Q2\;\times \;Q3}{{\left(\frac{Q1+Q2+Q3}{3}\right)}^{3}}} \end{array}$$

(9) $$\left\{ \begin{array}{l} T=\delta Q_{1}+\varepsilon Q_{2}+\vartheta Q_{3}\\ \;D=\sqrt{C\times T} \end{array}\right.$$

In Formulas (8) and (9), Q1, Q2, and Q3 denote the comprehensive evaluation index of LCD, TI, and NU subsystems. *C* is the coupling degree of the three subsystems, with the value domain space of [0, 1]. *D* is the coupling coordination degree. *T* is the comprehensive evaluation index. Their value domain space are both [0, 1]. δ,ε,ϑ are the coefficients to be determined, indicating the importance of the three subsystems. They satisfy δ+ε+ϑ = 1. In this paper, we consider LCD, TI and NU as equally important, so we make δ = ε = ϑ = *1/3*.

In order to intuitively represent the coupling coordination status of the three subsystems, the above CCD is divided into the following levels (Table 3).

**Table 3:** Coupling coordination degree level type of LCD quality/TI capability/NU level.

**Category**	**Coupling coordination value (D)**	**Coordination level**
Maladjusted	0.0 ≤ D ≤ 0.2	Severely maladjusted
	0.2 < D ≤ 0.3	Moderately maladjusted
	0.3 < D ≤ 0.4	Slightly maladjusted
General coordination	0.4 < D ≤ 0.5	On the verge of maladjustment
	0.5 < D ≤ 0.6	Barely coordinated
	0.6 < D ≤ 0.7	Primary coordination
Higher coordination	0.7 < D ≤ 0.8	Moderately coordinated
	0.8 < D ≤ 0.9	Good coordinated
	0.9 < D ≤ 1	Pre-eminently coordinated

### Exploratory spatial data analysis

In order to explore the spatial correlation characteristics of the coupling level of the “LCD-TI -NU” system in China, this paper conducts spatial autocorrelation test and analyze the spatial correlation between systems based on *Global Moran's I* index. The formula is as follows:


(10) $$\begin{array}{l} Global\;Moran^{\prime }s\;I\;=\frac{n\sum_{i=1}^{n}\sum_{j=1}^{n}w_{ij}\left(y_{i}\;-\bar{y}\right)\left(y_{j}-\bar{y}\right)}{\left(\sum_{i=1}^{n}\sum_{j=1}^{n}w_{ij}\right)\sum_{i=1}^{n}{\left(y_{i}-\bar{y}\right)}^{2}} \end{array}$$

In Formula (10), *I-value* indicates the global autocorrelation Moran index, which is used to determine whether there is a correlation between spatial entities in a certain range. The range of *I-value* is –*1* ≤ *I* ≤ *1, I* > *0* means positive spatial correlation, *I*<*0* means negative spatial correlation, and *I* = *0* means no correlation. *n* indicates the number of spatial units. *y*_*i*_ and *y*_*j*_ indicate the observed values of coupling coordination of province *i* and province *j*. *y* indicates the average value of coupling coordination of each province. *w*_*ij*_ is the distance spatial weight of provinces *i* and *j*, *w*_*ij*_ = *1* when *i* and *j* are adjacent, *w*_*ij*_ = *0* when *i* and *j* are not adjacent.

To further explore whether there is spatial heterogeneity, this paper performs a local spatial autocorrelation test based on the *Local Moran's I* index.


(11) $$\begin{array}{l} Local\;Moran^{\prime }s\;I_{i}=\frac{n\left(y_{i}-\bar{y}\right)\sum_{j=1,j\neq i}^{n}w_{ij}\left(y_{j}-\bar{y}\right)}{\sum_{i=1}^{n}{\left(y_{i}-\bar{y}\right)}^{2}} \end{array}$$

In Formula (11): *I*_*i*_ represents the local Moran's coefficient for the *i-th* region. *I*_*i*_ > 0 indicates that the high (low) value of *province-i* is surrounded by the high (low) values. *I*_*i*_ < 0 indicates that the high (low) value of *province-i* is surrounded by the low (high) values. *y*_*i*_ and *y*_*j*_ are observed values. $\bar{y}$ is the mean value. *w*_*ij*_ is the spatial weight value.

### Obstacle degree model

This paper hopes to identify the subsystems and indexes that have a significant impact on the CCD of “LCD-TI-NU”, with the aim of proposing reasonable optimization solutions for improving the synergistic development of the three subsystems in each region. The obstacle degree model is often used to identify the obstacles affecting things development (63) and specify key factors and effect degree. In this paper, this model is more operationally feasible and practically valuable given the relationship between the formulas. The formula is as follows.


(12) $$O_{ij}=\frac{\left(1-X_{ij}^{{}^{\prime }}\right)\times w_{j}}{\overset{m}{\underset{j}{\sum }}w_{j}\times \left(1-X_{ij}^{{}^{\prime }}\right)}$$

In Formula (12), *O*_*ij*_ represents the obstacle degree of the *j-th* index in the *i-th* sample. ${X{}^{ij}}_{\prime }$ is the standardized value of the *j-th* index in the *i-th* sample. The difference between *1* and ${X{}^{ij}}_{\prime }s$ indicates the difference between the actual value of the index and the optimal value. *w*_*j*_ is the weight of the index (64, 65).

## Empirical research and analysis of results

Based on the data of 30 China's provincial regions from 2009 to 2019, this paper calculated the comprehensive evaluation index of each subsystem using MATLAB software, and obtained the coupling degree and CCD of the three subsystems based on the coupling coordination model (Table 3). In order to explore the spatial differences and evolutionary characteristics of the coupling coordination, this paper analyzed and examined the spatial autocorrelation of the data with the help of ArcGIS and GeoDa software.

### Comprehensive evaluation index analysis of the LCD/TI/NU subsystem

**Figure 1:**
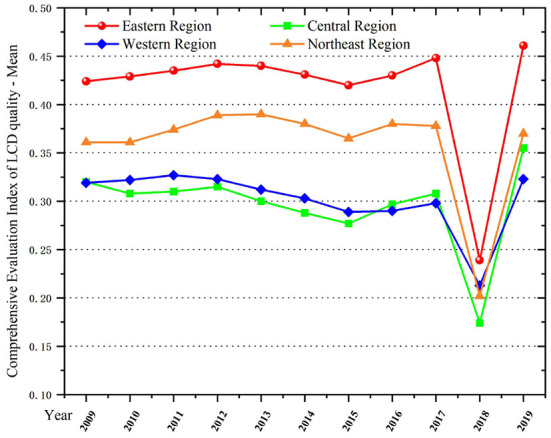
Comprehensive evaluation index of LCD quality in four regions of China, 2009–2019.

As shown in Figure 1, the region's LCD quality shows a slow improvement and up-and-down fluctuation from 2009 to 2019. During this period, the LCD quality of the eastern region is significantly superior to other regions, followed by the northeast region. LCD quality of the central and western regions is the lowest. Compared with the eastern region, the northeast region's industrialization shows a continuous trend of expansion. Economic growth is heavily dependent on the secondary industry, resulting in high energy consumption in the region. However, there are more traditional industries and resource-consuming industries in the central and western regions, which lead to large consumption of electricity and water resources. Meanwhile, due to their relatively backward awareness of green development, the LCD quality of these two regions is not good enough. In 2018, the comprehensive evaluation index of LCD reached an inflection point, and it declined sharply in various regions. In terms of the international situation, global carbon emissions from energy consumption grew in 2018 at the fastest rate since 2010. At the same time, many of the world's major energy consumers, particularly the United States, China and Russia, have experienced large amounts of unusual weather, which has boosted demand for heating or cooling. Meanwhile, China's energy-intensive industries were undergoing transformation in 2018. The cyclical nature of industrial transformation has led to a rebound in energy demand and consumption. Moreover, while population density has increased, infrastructure development has lagged slightly. These factors directly contributed to a further decline in the quality of LCD across the country in 2018, marking an inflection point. On the whole, the regions with high qualities of LCD include Beijing, Guangdong, Shanghai and Jiangsu. These regions have a relatively high level of economic superiority and rapid industrial upgrading. The tertiary industry in these regions accounts for a high proportion, and the supporting service industry is relatively well developed. Therefore, they are outstanding in the efficient use of resources, energy and environmental pollution control. Also, these regions have stricter enforcement standards on emission targets so that they emit less pollutants and have obvious advantages in LCD.

**Figure 2:**
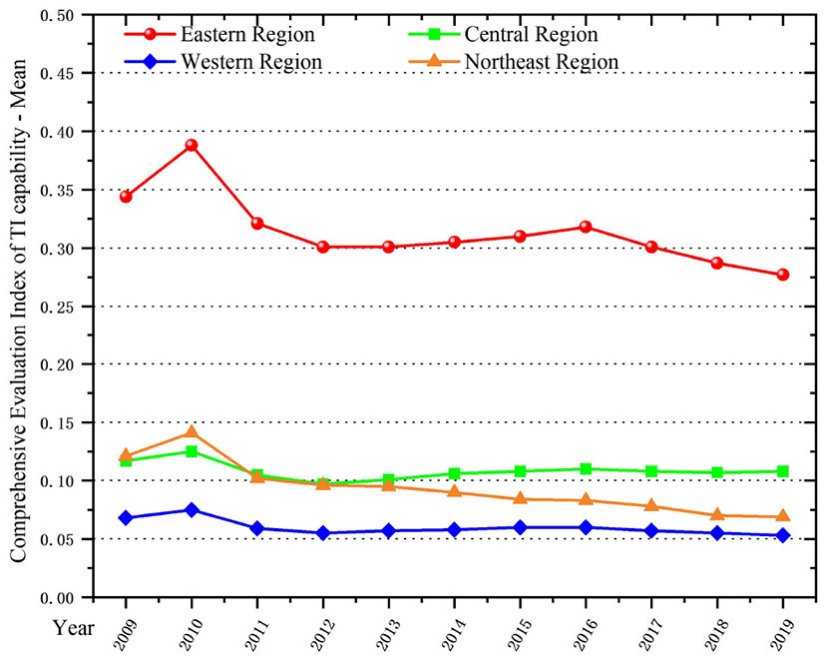
Comprehensive evaluation index of TI capability in four regions of China, 2009–2019.

In terms of the comprehensive development level of TI (Figure 2) from 2009 to 2019, the national level of TI has been continuously improved, and the TI capability in the eastern region is far ahead of other regions, showing a fault-type leading situation. The reason for this is the high level of economic development in eastern regions. Furthermore, the concentration of qualified technical talents and the increased number of high-tech enterprises lead to the abundance of innovation energy in the region. The western region, on the other hand, has a low overall innovation capacity due to the limited innovation environment, innovation resources and insufficient transformation of results in most provinces. Several provinces and cities with more prominent TI capabilities mainly include: Beijing, Guangdong, Jiangsu, Sichuan and Shaanxi.

**Figure 3:**
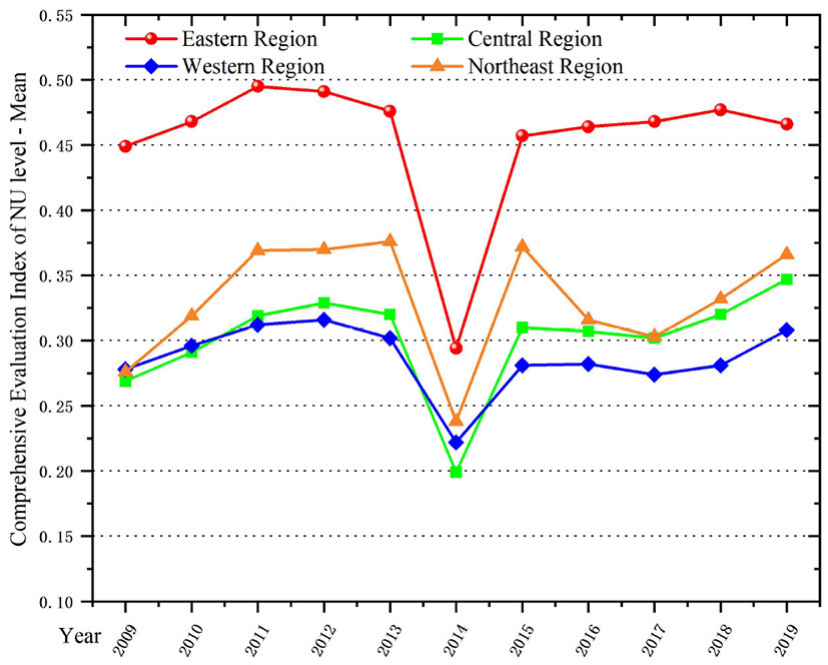
Comprehensive evaluation index of NU level in four regions of China, 2009–2019.

In terms of the comprehensive development level of NU (Figure 3), the eastern region is still ahead of other regions, followed by the northeast region. The reason is that the northeast region of China covers most of the heavy industrial industries. These places have a higher level of industrialization, more developed railroad transportation and higher urbanization rates. In 2014, the NU level in all regions declined sharply and reached an inflection point. According to the observation data analysis, we found that in December 2013, the government made the first attempt to implement the *National New Type Urbanization Plan (2014–2020)*, and carried out a series of comprehensive pilot projects nationwide. However, due to the initial stage, each region was facing multiple pressures. Population urbanization took precedence over land urbanization, and regions expanded industries to absorb more people, which also led to extremely prominent environmental pollution problems as a result. Moreover, the registered unemployment rate in urban areas has increased significantly. It was particularly prominent in developed regions and neighboring provinces. Since 2015, the level of NU in various regions has begun to rise. The growth rate of NU was very fast in central and western regions, such as Guizhou, Yunnan, Gansu, Guangxi and Ningxia provinces. These regions had relatively low urbanization rates due to their mountainous terrain with little flat land, lagging education development and late start of industrialization. However, it has shown a trend of steady increase in recent years.

### Spatial-temporal characteristics of the “LCD-TI-NU” system

**Figure 4:**
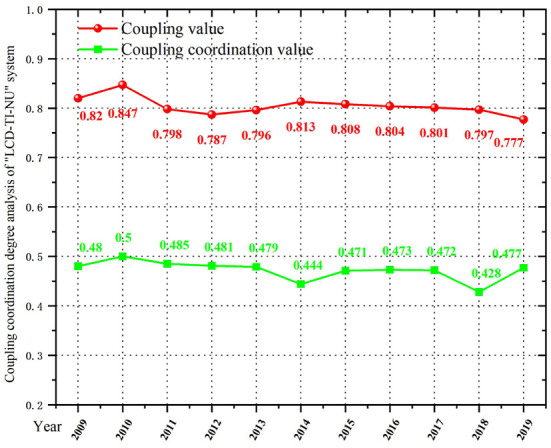
Coupling coordination degree analysis of “LCD-TI-NU” system in China, 2009–2019.

As shown in Table 4 and Figure 4, the mean values of coupling degree and CCD of “LCD-TI-NU” system from 2009 to 2019 is 0.804 and 0.472, respectively. This indicates that the LCD quality, TI capability and NU level in China are at a high level of synchronization, showing an obvious interaction relationship. However, due to the low level of comprehensive development of each subsystem and the differences between regions, the overall CCD is not high. This further shows that there is still a huge potential for system optimization.

**Table 4:** Coupling coordination degree of the “LCD-TI-NU” system, 2009–2019.

**Region**	**Province**	**2009**	**2010**	**2011**	**2012**	**2013**	**2014**	**2015**	**2016**	**2017**	**2018**	**2019**
Eastern region	Beijing	0.813	0.765	0.805	0.802	0.812	0.762	0.820	0.816	0.805	0.707	0.785
	Tianjin	0.522	0.551	0.539	0.549	0.559	0.515	0.550	0.551	0.547	0.475	0.507
	Hebei	0.445	0.468	0.446	0.436	0.438	0.404	0.442	0.443	0.446	0.403	0.457
	Shanghai	0.674	0.680	0.664	0.662	0.649	0.608	0.650	0.650	0.654	0.569	0.621
	Jiangsu	0.711	0.742	0.717	0.724	0.718	0.660	0.705	0.712	0.701	0.626	0.687
	Zhejiang	0.677	0.700	0.672	0.651	0.652	0.599	0.642	0.649	0.646	0.591	0.656
	Fujian	0.512	0.537	0.512	0.506	0.504	0.458	0.494	0.496	0.502	0.465	0.514
	Shandong	0.572	0.613	0.584	0.580	0.576	0.517	0.553	0.566	0.568	0.505	0.562
	Guangdong	0.725	0.765	0.740	0.723	0.710	0.653	0.702	0.732	0.745	0.677	0.751
	Hainan	0.290	0.318	0.321	0.317	0.308	0.277	0.301	0.299	0.297	0.276	0.308
	Mean	0.594	0.614	0.600	0.595	0.592	0.545	0.586	0.591	0.591	0.529	0.585
Central region	Shanxi	0.385	0.387	0.390	0.389	0.397	0.365	0.390	0.395	0.374	0.343	0.395
	Anhui	0.457	0.460	0.474	0.472	0.474	0.434	0.466	0.466	0.474	0.435	0.500
	Jiangxi	0.432	0.457	0.428	0.422	0.417	0.378	0.408	0.420	0.436	0.396	0.449
	Henan	0.466	0.469	0.463	0.464	0.454	0.427	0.462	0.472	0.474	0.437	0.492
	Hubei	0.514	0.525	0.521	0.513	0.510	0.471	0.502	0.511	0.506	0.464	0.519
	Hunan	0.485	0.498	0.480	0.474	0.476	0.446	0.477	0.478	0.482	0.447	0.524
	Mean	0.457	0.466	0.460	0.456	0.454	0.420	0.451	0.457	0.458	0.420	0.480
Western region	Inner Mongolia	0.348	0.374	0.360	0.365	0.371	0.340	0.363	0.370	0.356	0.308	0.338
	Guangxi	0.404	0.440	0.392	0.385	0.375	0.340	0.360	0.359	0.373	0.341	0.377
	Chongqing	0.431	0.461	0.467	0.471	0.464	0.508	0.458	0.457	0.459	0.419	0.473
	Sichuan	0.505	0.496	0.512	0.514	0.510	0.467	0.493	0.494	0.501	0.465	0.524
	Guizhou	0.338	0.379	0.352	0.348	0.340	0.313	0.341	0.338	0.337	0.303	0.347
	Yunnan	0.404	0.444	0.405	0.392	0.377	0.353	0.390	0.392	0.388	0.349	0.398
	Shaanxi	0.553	0.535	0.549	0.541	0.542	0.497	0.518	0.519	0.505	0.461	0.526
	Gansu	0.373	0.390	0.377	0.385	0.374	0.346	0.372	0.372	0.357	0.318	0.356
	Qinghai	0.291	0.315	0.289	0.280	0.272	0.248	0.237	0.218	0.248	0.208	0.248
	Ningxia	0.312	0.333	0.309	0.304	0.308	0.287	0.306	0.297	0.315	0.289	0.331
	Xinjiang	0.341	0.385	0.325	0.313	0.319	0.304	0.330	0.324	0.304	0.358	0.299
	Mean	0.391	0.414	0.394	0.391	0.387	0.364	0.379	0.376	0.377	0.347	0.383
Northeast region	Liaoning	0.516	0.523	0.533	0.526	0.524	0.472	0.503	0.497	0.496	0.440	0.487
	Jilin	0.431	0.458	0.437	0.440	0.437	0.399	0.416	0.406	0.395	0.362	0.412
	Heilongjiang	0.477	0.521	0.488	0.492	0.499	0.463	0.495	0.477	0.464	0.415	0.467
	Mean	0.474	0.501	0.486	0.486	0.487	0.445	0.471	0.460	0.451	0.406	0.455

From the perspective of time evolution, the year of 2014 is the cut-off point for the system's CCD of China's regions. Before 2014, it showed a state of fluctuation, and after 2014, the overall CCD showed a slow downward trend. Combined with the comprehensive evaluation index, this paper finds that the comprehensive development level of TI subsystem fluctuates, and the overall trend is declining. The indexes are reflected in the *Number of R&D Institutions*, the *Full-Time Equivalent of R&D Personnel by Region*, and the *Decrease of Expenditure on New Products Development*. Also, the differences between regions are distinct. At the same time, the overall coupling coordination pattern of provinces and cities has obvious gap and is extremely unbalanced. During the study period, the overall CCD of LCD quality, TI capability and NU level in each region are still in the breaking-in period. Turning points occurred in 2014 and 2018. Generally speaking, subsystem's development level is related to the coupling coordination level of the whole system. In 2014, the overall CCD dropped sharply from 0.479 to 0.444 due to the short lag of the NU subsystem. Similarly, in 2018, the LCD subsystem faced the increasing energy consumption and pollution deterioration, which led to the low comprehensive evaluation index, and finally affected the coupling coordination development of the three subsystems. The CCD decreased from 0.472 to 0.428 during the year, reaching the lowest value in the study period. In order to improve the coordinated development level of “LCD-TI-NU” system among regions, it is necessary to emphasize the integrity and synergy of relevant policies and measures, and promote the overall optimization on the basis of improving the comprehensive level of each subsystem.

**Figure 5:**
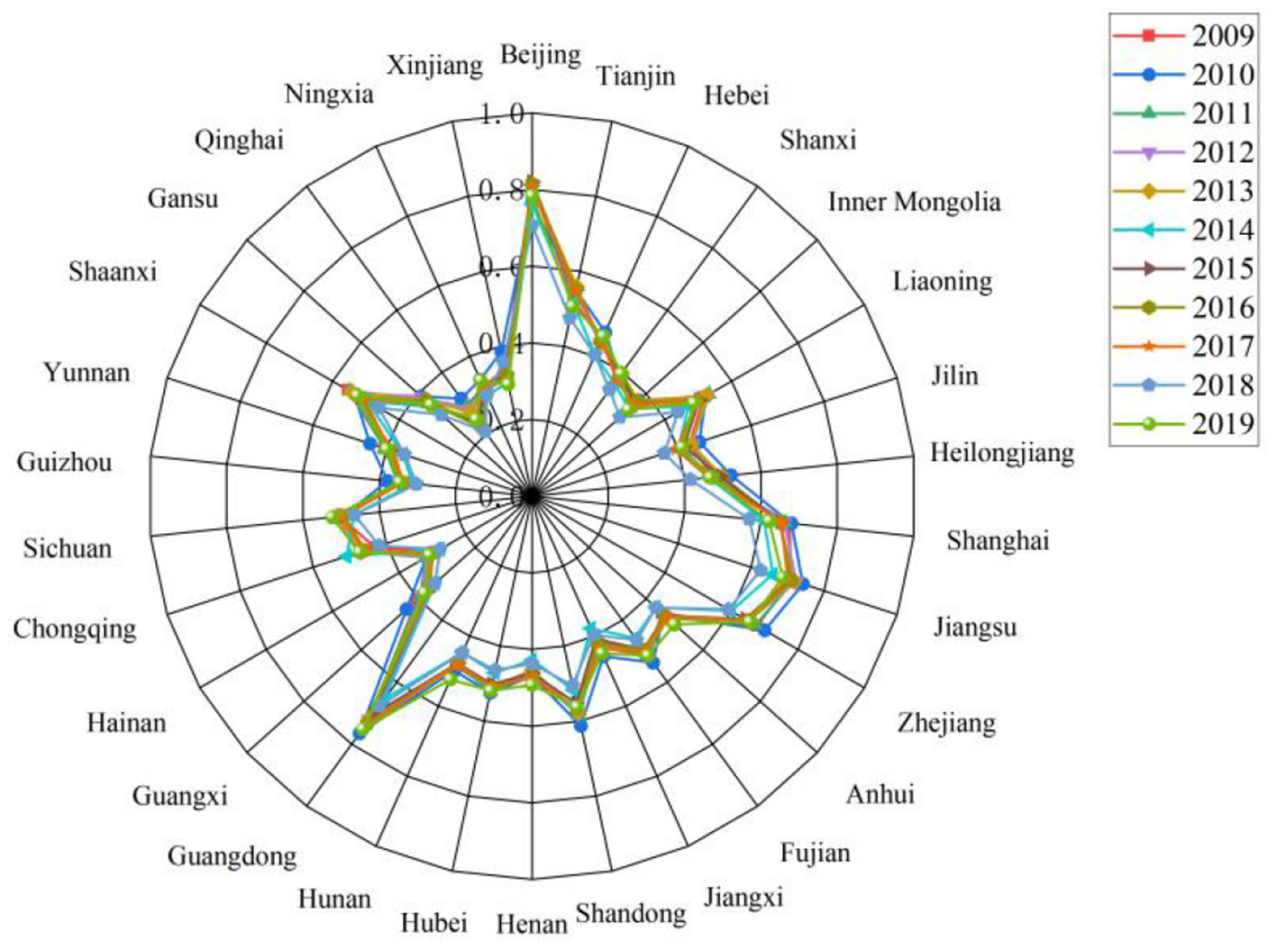
Coupling coordination degree of “LCD-TI-NU” system in China's provinces and cities, 2009–2019.

**Figure 6:**
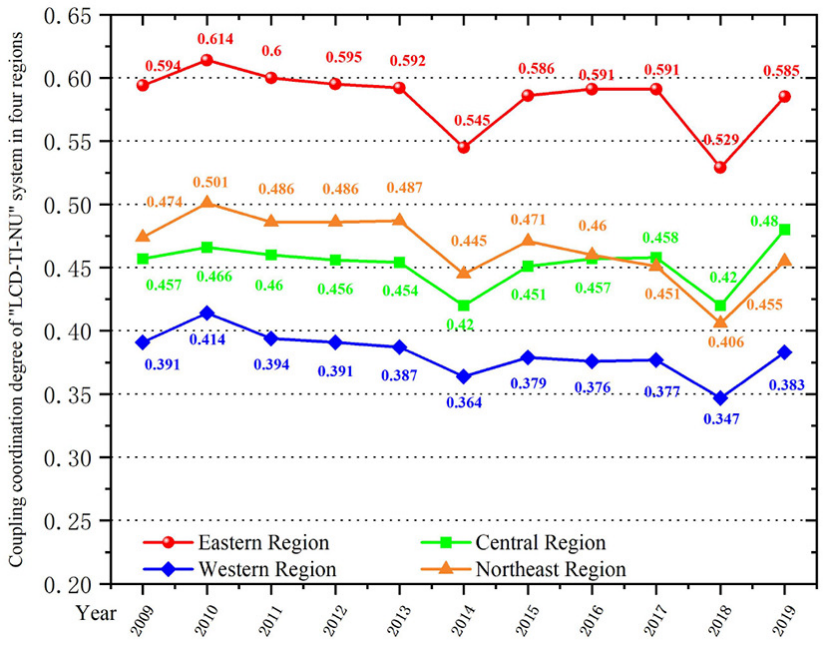
Coupling coordination degree analysis of “LCD-TI-NU” system in four regions, 2009–2019.

From the perspective of spatial distribution (Figures 5, 6), the coupling coordination level of the three subsystems in China is gradually decreasing in the eastern, northeast, central and western regions. Specifically, the CCD in the eastern region fluctuates from 0.529 to 0.614, and is always higher than the national average level, reaching the level of barely coordination. Among them, Beijing, Shanghai, Jiangsu and Guangdong perform better. These four regions show a good coordination trend, followed by Zhejiang and Shandong provinces, which maintain the primary coordination level. The average level of coupling coordination between northeast and central regions is about the same, and the CCD is concentrated around 0.45, which belongs to the verge of maladjustment. In central regions, with the exception of Hubei Province in some years (2014 and 2018), the CCD is above 0.5, which is at the level of barely coordinated. The CCD of Hunan Province and Anhui Province shows a slow upward trend, reaching 0.5 in 2019, from the level of near maladjustment to the barely coordinated level. In western regions, the CCD of almost all provinces and cities is lower than 0.5 except Shaanxi Province and Sichuan Province, which reach the barely coordinated level. The CCD of almost all regions is about 0.3 and 0.4. This means that the LCD quality, TI capability and NU level in these regions are in a slightly maladjusted level, which needs to be paid attention to. As the TI capability of the western region is significantly lower than that of other regions, the TI capability lags behind the LCD quality and NU process, resulting in a large gap between subsystems. This also leads to the lowest CCD in western regions. Although in recent years, the country has a certain policy tilt and encouragement measures to the western regions so that TI capability has been improved to a certain extent. However, it is still in a backward position in the whole country. This requires these western regions to balance the relationship between LCD, TI and NU. Moreover, attention should be paid to the role of TI capability in realizing low-carbon environmental protection development and smart city construction.

**Figure 7:**
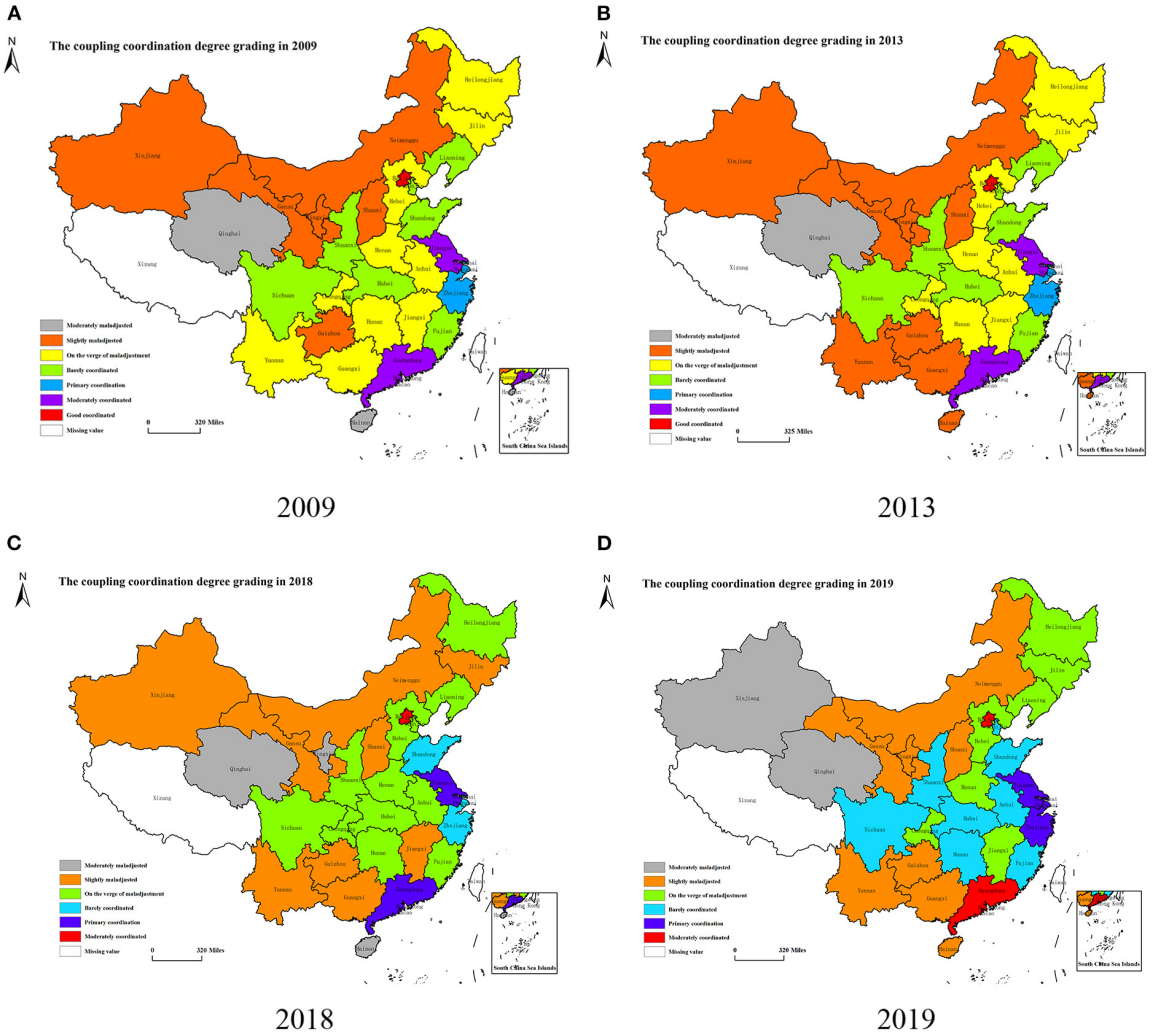
**(A–D)** Spatial distribution of coupling coordination degree of “LCD-TI-NU” system in some years.

From the perspective of spatial pattern (Table 5; Figure 7), there is spatial and temporal variability in the coupling coordination levels of “LCD-TI-NU” system. In 2010, the CCD is divided into five grades: “*slightly maladjusted*” to “*moderately coordinated*”. In the year of 2014, 2018, and 2019, it is divided into 6 grades from “*moderately maladjusted*” to “*moderately coordinated*”. The CCD's span of the other years is from “*moderately maladjusted*” to “*good coordinated*”, a total of 7 grades. This also indicates that there are huge regional differences in the coupling coordinated development level of this system. In Table 5, from 2009 to 2013, the regions with CCD > 0.5 (that is, reaching the coordinated level) accounted for 40%. It fell to 26% in 2014 and 20% in 2018, and then increased to 43% in 2019. It can be seen that the increase of the overall CCD is not high, but the regions that reach the level of coordinated level show a slow upward trend. In Figure 7, the spatial characteristics in 2009, 2013, 2018, and 2019 are more representative. It can be seen from this that from 2009 to 2018, the regions on the verge of maladjustment do not show a trend of leapfrog to the direction of barely coordinated, but show a trend of malignant development. For example, Yunnan, Guangxi and Jiangxi all enter the grade of slightly maladjusted in 2018 (Figure 7C). In 2019 (Figure 7D), the situation begins to improve, with the Sichuan, Shaanxi, Hubei, Hunan, Fujian and Anhui provinces all reaching the barely coordinated level. Guangdong Province also enters the moderately coordinated level. In terms of spatial pattern, the provinces with coupling maladjustment are mainly concentrated in the western region, while the central and northeast regions are mainly on the verge of maladjustment and barely coordinated, and there is still a lot of room for improvement. The eastern region has the highest overall level of coordinated development, with the exception of Hainan, Hebei and Fujian in some years, all provinces have reached the primary coordination level. Furthermore, the standard deviation of the CCD of 30 provincial administrative regions in China is 0.1337 in 2009 and decrease to 0.1312 in 2019, while the standard deviation reflects the degree of dispersion of the sample data. The decrease in standard deviation indicates a tendency for the differences between provinces to narrow.

**Table 5:** Number of provinces and cities with different coupling coordination levels in China, 2009–2019 (unit).

**Classification**	**2009**	**2010**	**2011**	**2012**	**2013**	**2014**	**2015**	**2016**	**2017**	**2018**	**2019**
Good coordinated	1	0	1	1	1	0	1	1	1	0	0
Moderately coordinated	2	3	2	2	2	1	2	2	2	1	2
Primary coordination	2	3	2	2	2	3	2	2	2	2	3
Barely coordinated	7	6	7	7	7	4	5	4	6	3	8
On the verge of maladjustment	10	10	9	8	8	10	10	11	8	12	7
Slightly maladjusted	6	8	8	9	9	9	9	7	9	9	8
Moderately maladjusted	2	0	1	1	1	3	1	3	2	3	2

### Spatial correlation analysis of the “LCD-TI-NU” system

#### Global spatial autocorrelation

The results show that the Moran's I of the coupling coordination levels are all significantly positive with *P* < 0.01 and *Z* > 2.5 during the period 2009–2019 (Table 6). It indicates that there is a significant positive correlation between the three subsystems and shows a significant agglomeration effect. The CCD is higher in the surrounding areas of areas with high CCD. Moran's *I-* and *Z*-values show a fluctuating increase with the passage of time. This further indicates that the system's spatial agglomeration degree is increasing.

**Table 6:** Results of the Global Moran's *I-*test.

**Variable**	**2009**	**2010**	**2011**	**2012**	**2013**	**2014**	**2015**	**2016**	**2017**	**2018**	**2019**
Moran's *I*	0.321	0.359	0.328	0.327	0.330	0.317	0.338	0.341	0.376	0.330	0.365
*Z*-value	2.763	3.050	2.812	2.804	2.828	2.733	2.910	2.930	3.192	2.834	3.101
*P*-value	0.006	0.002	0.004	0.005	0.005	0.006	0.004	0.003	0.001	0.005	0.002

#### Local spatial autocorrelation

This paper used local Moran's I index scatter plots with LISA agglomerative plots in typical years (2009/2010/2019) to reflect the local situation of spatial linkage. The scatter plot represented the observed values' linear combination in any region and the observed values in other regions.

**Figure 8:**
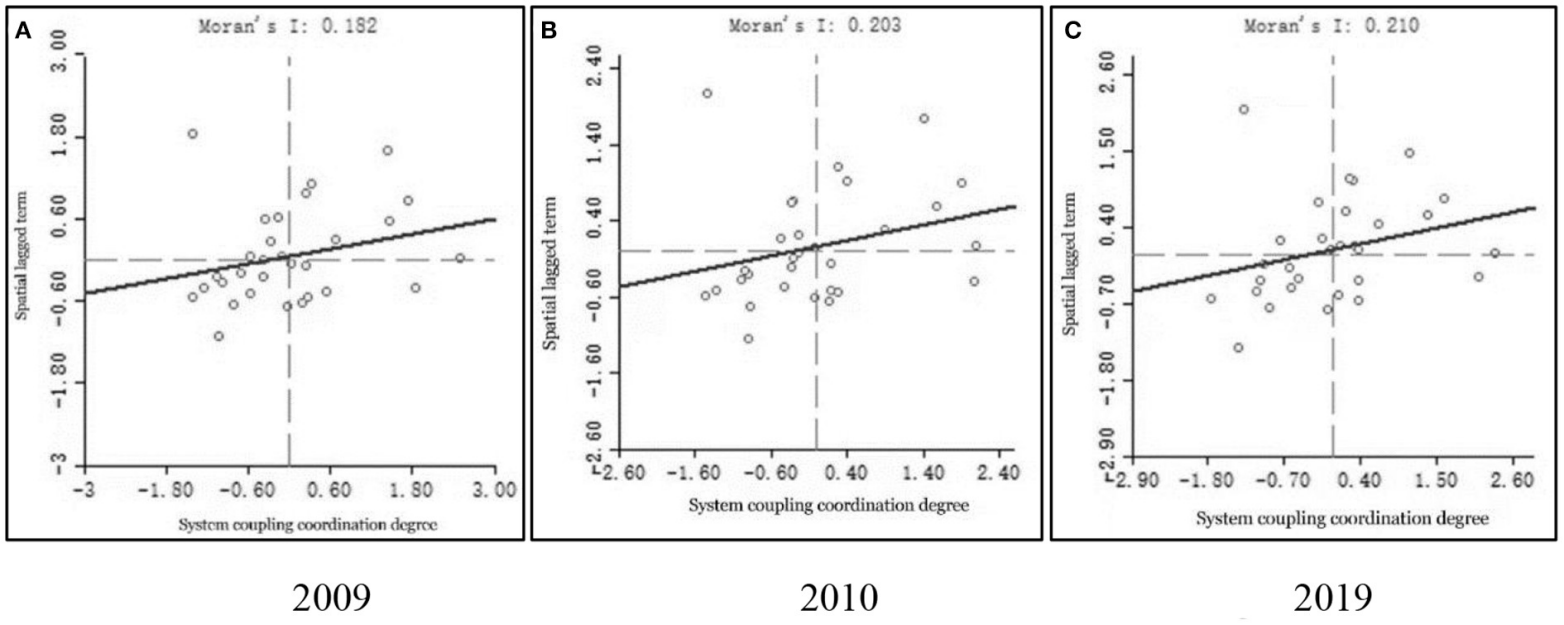
**(A–C)** Moran scatter plot of coupling coordination degree in 2009, 2010, and 2019.

In Figure 8, the horizontal axis represents the standardized data (observed values) of CCD in province-*i*, and the vertical axis represents the linear combination's observed values of CCD in other regions. The quadrants one and three in the coordinate axes indicate the clustering of high-high values and low-low values, respectively, which represent positive spatial autocorrelation and indicate the clustering of similar values. On the contrary, quadrants two and four represent the clustering of low-high value (Province-*i* is low value, but all surrounding areas are high value) and high-low (Province-*i* is high value, but all surrounding areas are low value), respectively, indicating that there is negative spatial autocorrelation in this region, which is a spatial anomaly. In Figures 8A–C, the local Moran's *I* in 2009, 2010, and 2019 are 0.182, 0.203, and 0.210, and the *P*-values are < 0.05, which are statistically significant. Also, the numbers of scattered points falling into quadrants one and three are 18, 21, and 20, with proportions of 60, 70, and 66.7%, respectively. Overall, the spatial correlation of the CCD of the “LCD-TI-NU” system in Chinese provinces shows an increasing trend during the study period, with up and down fluctuations in a few years.

**Figure 9:**
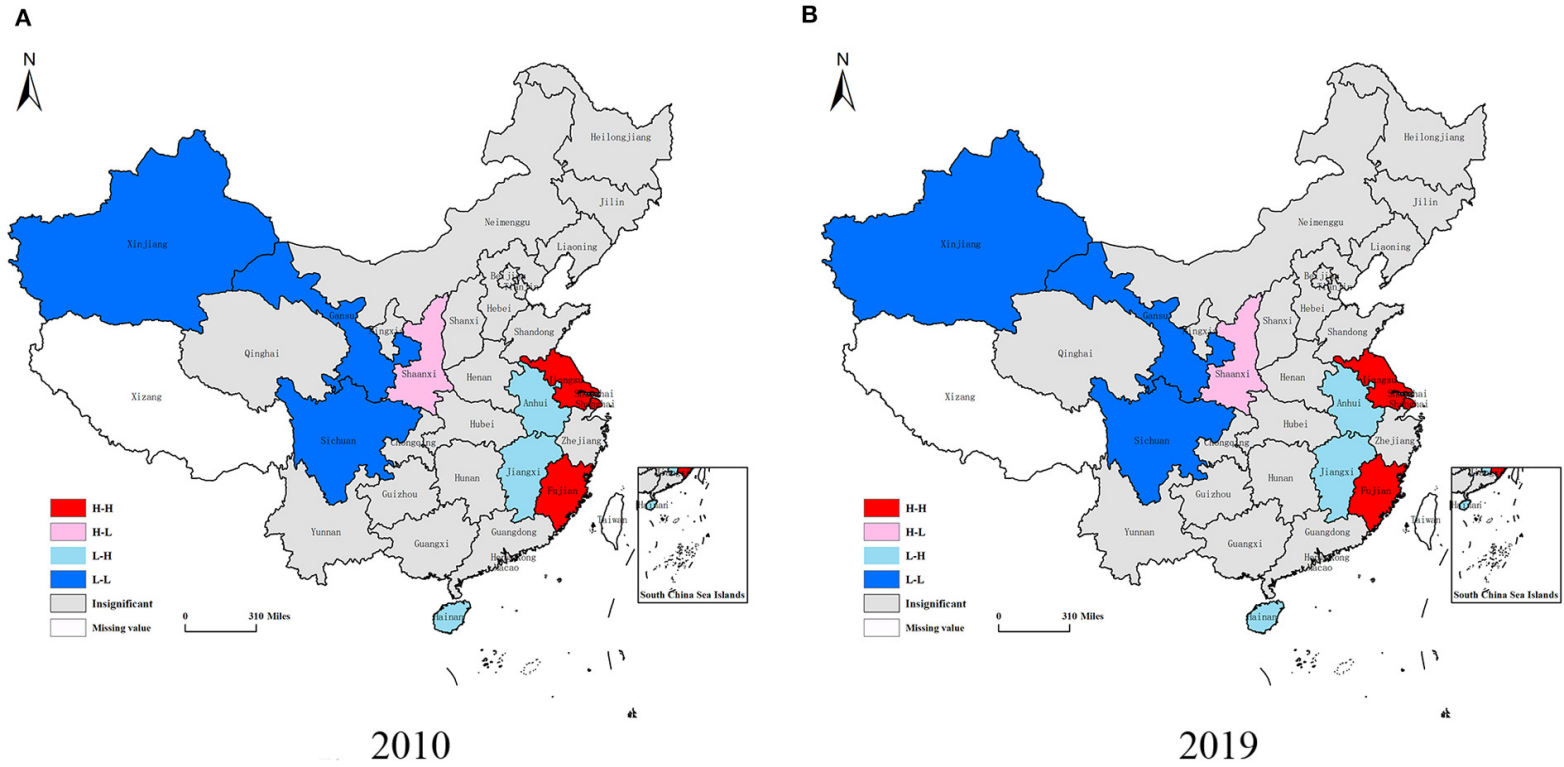
**(A,B)** LISA agglomerative plot of system coupling coordination degree in 2010 and 2019.

As shown in Figures 9A,B, the provinces with high CCD are mostly concentrated in the eastern regions, such as Jiangsu, Fujian and Shanghai. The regions with low values are mainly in the western regions of Gansu, Sichuan and Xinjiang. The main representative provinces of high-low agglomeration are Shaanxi Province in 2010 and Sichuan Province in 2019. These two regions are among the provinces with high CCD, but their surrounding areas are low-value provinces. Low-high agglomeration is exemplified by Hainan and Jiangxi provinces, which have a low CCD of their own, but their neighboring provinces such as Guangdong, Hubei and Fujian are high-value provinces. The state of positive spatial correlation (H-H and L-L) has increased. The increase of coupling coordination level in the eastern region will lead to the improvement of the overall situation in the central and western regions in turn.

### Identification of obstacle factors in the “LCD-TI-NU” system

**Figure 10:**
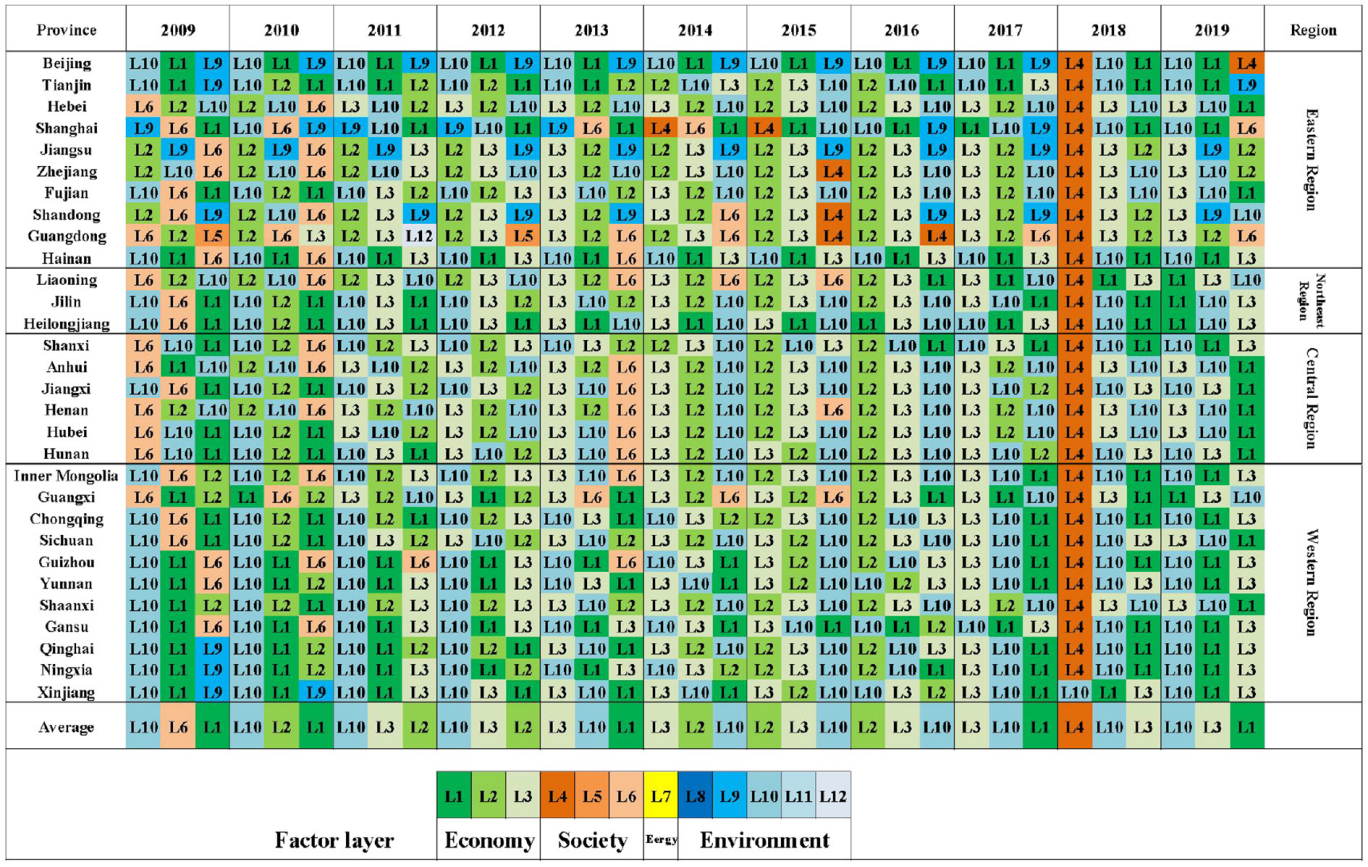
Top three obstacle indexes of LCD subsystem in different regions, 2009–2019.

**Figure 11:**
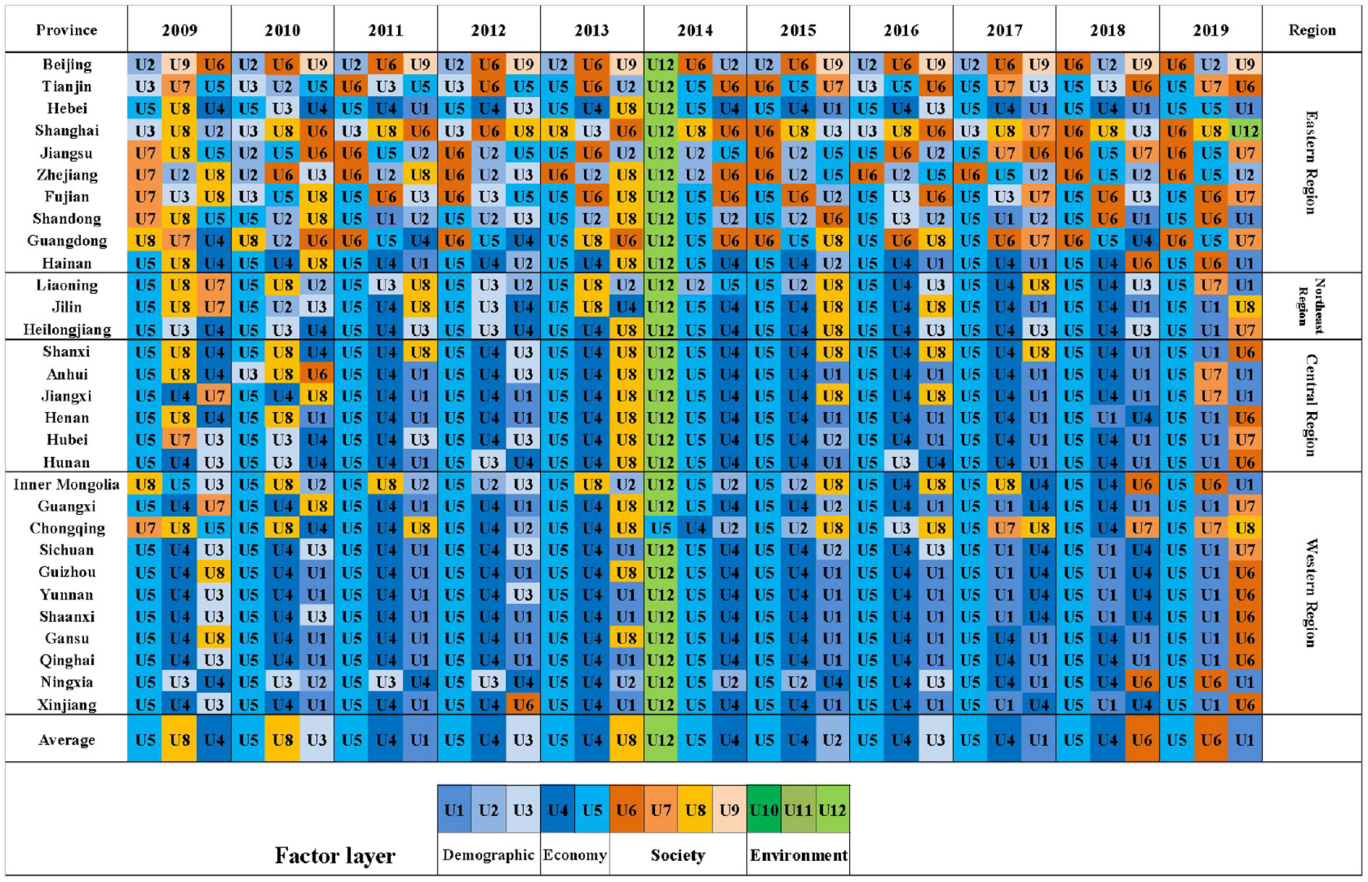
Top three obstacle factors of NU subsystem in different regions, 2009–2019.

The obstacle factors affecting the synergetic development of the three subsystems are shown in Table 7. In this paper, the first three indexes of obstacle degree are selected. Figures 10, 11 list first three indexes in terms of obstacle degree for each province and city each year of LCD quality and NU level, respectively. There are differences in the color of the indexes at different layers, which facilitates the visual clarification of the main factor layer of the obstacle factors in different regions. The regional variability of index obstacle degree in TI subsystem is not significant, so the obstacle indexes for each region are not shown separately.

**Table 7:** Obstacle indexes of each subsystem, 2009–2019.

	**LCD subsystem**			**TI subsystem**			**NU subsystem**		
	**1st**	**2nd**	**3rd**	**1st**	**2nd**	**3rd**	**1st**	**2nd**	**3rd**
2009	L10 (16.1%)	L6 (15.7%)	L1 (14.6%)	T7 (20.9%)	T8 (20.1%)	T6 (15.7%)	U5 (15.5%)	U8 (13.7%)	U4 (12.8%)
2010	L10 (17.6%)	L2 (15.3%)	L1 (13.8%)	T7 (23.6%)	T6 (17.3%)	T3 (16.6%)	U5 (16.8%)	U8 (12.5%)	U3 (12.3%)
2011	L10 (16.5%)	L3 (13.8%)	L2 (13.1%)	T7 (20.4%)	T8 (19.3%)	T6 (16.0%)	U5 (18.5%)	U4 (12.6%)	U1 (11.7%)
2012	L10 (16.4%)	L3 (15.0%)	L2 (14.2%)	T7 (19.4%)	T8 (17.8%)	T3 (17.2%)	U5 (18.8%)	U4 (13.4%)	U3 (13.4%)
2013	L3 (16.0%)	L10 (14.9%)	L2 (12.7%)	T8 (19.1%)	T3 (19.0%)	T7 (18.9%)	U5 (18.9%)	U4 (13.8%)	U8 (13.7%)
2014	L3 (17.1%)	L2 (15.5%)	L10 (14.4%)	T7 (20.2%)	T8 (19.7%)	T3 (19.6%)	U12 (47.4%)	U5 (10.1%)	U4 (8.4%)
2015	L2 (19.1%)	L3 (18.7%)	L10 (13.3%)	T7 (20.7%)	T3 (20.0%)	T8 (19.6%)	U5 (18.1%)	U4 (12.6%)	U2 (12.4%)
2016	L2 (20.0%)	L3 (15.1%)	L10 (14.9%)	T3 (21.9%)	T7 (21.2%)	T8 (19.3%)	U5 (17.9%)	U4 (12.6%)	U3 (11.6%)
2017	L3 (19.1%)	L10 (16.3%)	L1 (14.4%)	T3 (23.1%)	T7 (21.1%)	T8 (17.9%)	U5 (18.5%)	U4 (12.1%)	U1 (11.9%)
2018	L4 (59.9%)	L10 (7.7%)	L3 (7.0%)	T3 (23.8%)	T7 (20.5%)	T8 (17.4%)	U5 (18.9%)	U6 (13.5%)	U4 (12.6%)
2019	L10 (18.2%)	L3 (16.6%)	L1 (16.5%)	T3 (23.5%)	T7 (21.0%)	T8 (17.9%)	U5 (21.3%)	U6 (14.4%)	U1 (13.2%)

1st, 2nd, and 3rd indicate the first, second and third obstacle factors of each subsystem, respectively.

In the LCD subsystem (Table 7), the highest barrier rate in 2009–2012 and 2019 is the *Daily Disposal Capacity of City Sewage* (L10), and this index is found in the first three for each year. This is followed by *Secondary Industry/GDP* (L2), *Tertiary Industry/GDP* (L3) and *Gross Regional Product* (L1). *Environmental Protection Expenditure/GDP* (L4) is the most influential index in 2018 with a value of 59.9%. This result is consistent with the sharp decline and inflection point of the comprehensive evaluation index of the LCD in 2018. The situation starts to improve in 2019, with the obstacle degree of L4 dropping to 8.3%. From Figure 10, it can be seen that the indexes affecting the LCD quality are mainly at the economic level (L1-L3), followed by the environmental indexes (L8-L12). Spatially, the eastern and central regions are more affected by the social dimension (L6). The main obstacle factors in the central and western regions are roughly the same as the average. The eastern regions are less affected by the economic dimension and more by the environment with their superior economic and trade advantages.

In the TI subsystem (Table 7), before the year of 2012, it was strongly influenced by the *Domestic Patent Granted by Region* (T6). Since 2012, the indexes that negatively affect the comprehensive development level of TI subsystem are mainly the *Expenditure on New Products Development* (T7), *Intramural Expenditure On R&D by Region* (T8) and *Number Of R&D Institutions* (T3). The first three obstacle factors in most areas are consistent with the average level. In eastern regions, such as Jiangsu Province and Guangdong Province, T1 and T5 have a negative impact on the improvement of local TI capacity in different years. Beijing and Shanghai are more influenced by T6.

Among the indexes that hinder the improvement of the comprehensive level of the NU subsystem (Table 7), the highest obstacle is the *Per Capita Disposable Income in Urban Area* (U5), followed by *Per Capita GDP* (U4). In 2009–2013, the obstacle degree of U8 and U3 are higher. In recent years, the obstruction degree of U6 andU1 also increased year by year. The obstacle degree of *Green Covered Area Rate of Completed Area* (U12) was 47.4% in 2014, indicating that U12 is lower in all regions compared to the rest of the years and had a more negative impact compared to the other indexes. This also directly leads to the lowest comprehensive evaluation index of NU in 2014. In Figure 11, the main obstacle indexes are concentrated in the economic (U4–U5) and population urbanization dimensions (U1–U3), followed by the social dimension (U6–U9). NU is less influenced by environmental indexes. Furthermore, the obstacle indexes of NU have obvious spatial variability. In eastern regions, the indexes of the social dimension are significantly more than those of the economic dimension. It indicates that compared with other regions, NU in the eastern region focuses more on people's livelihood and basic public service level due to its higher economic level. The central and western regions, on the other hand, are more influenced by economic indexes. In terms of temporal evolution, the obstacle degree of social indexes has gradually increased in recent years, which indicates the growing demand of urban residents for quality public services and social security, etc. However, the current growth rate of investment in livelihood expenditure is low compared to other aspects. In order to create a more harmonious social ecology, the central and western regions should steadily promote infrastructure development on the basis of vigorous economic development. The eastern and northeast regions can expand the investment in public services to improve the quality of life of their people.

## Discussion and conclusion

### Conclusions

Since the synergistic development relationship between the three subsystems has not yet been clarified, this paper firstly constructed the evaluation index system of LCD quality, TI capability and NU level in combination with current development requirements. Secondly, this paper calculated and analyzed the data based on the entropy method and the coupling coordination model. Subsequently, the comprehensive evaluation index, coupling degree and coupling coordination degree of the three systems in different years of each region were clarified. Finally, this paper conducted descriptive statistics and spatial correlation analysis. On this basis, the obstacle indexes that affected the comprehensive evaluation index of each subsystem were found out by using the obstacle degree model, which also provided ideas for improving the coupling coordination between systems. The result is as follows:

(1) There are regional imbalances in the level of LCD, TI, and NU in China's provinces. The eastern region has attracted a large number of people to gather with its geographical advantages and strong development strength. This has promoted NU level and TI capability locally, and the LCD quality has continued to lead. The comprehensive evaluation index of TI in the eastern region is far ahead of other places. Among them, Jiangsu, Guangdong and Beijing perform more prominently, followed by Zhejiang, Shanghai and Shandong. All of the above are in the eastern region. Sichuan and Shanxi provinces in the western region, and Hubei and Anhui provinces in the central region are behind. The TI capability of central regions has been increasing year by year, while other regions have fluctuated and declined slightly. The overall level of NU gradually increased from 2009 to 2012, decreased in 2013, and then began to slowly increase in 2014. Regionally, the growth rate of NU in northeast regions is significantly higher than that in other regions. The central and western region's growth rate ranks second, while the growth rate of eastern regions is relative to the minimum. The LCD quality in various regions shows a steady improvement in general, and the trend of changes between regions is in the same direction.
(2) The CCD of LCD quality, TI capability and NU level in various regions have been in a state of fluctuation for a long time, and the trend of increase and decrease is not obvious. This means that the regions are in a breaking-in period. This is due to the unsynchronized development of the three subsystems in various regions. The discrepancy between regions and systems is apparent, resulting in fluctuations in the trend of coupling coordination. Nevertheless, the overall development trend of the CCD of these three subsystems is ideal. In 2019, the proportion of provincial regions whose average CCD meets the coordination requirements (*D*-value > 0.5) reaches 43.3% (Beijing, Guangdong, Jiangsu, Zhejiang, Shanghai, Shandong, Shaanxi, Sichuan, Hunan, Hubei, Fujian, Tianjin, and Anhui). 23.3% of the regions face a crisis of near-maladjustment (Henan, Liaoning, Chongqing, Heilongjiang, Hebei, Jiangxi, and Jilin). 26.7% of the regions are slightly maladjusted (Yunnan, Shanxi, Guangxi, Gansu, Guizhou, Inner Mongolia, Ningxia, and Hainan), while 6.7% of the regions are moderately maladjusted (Xinjiang and Qinghai). Moreover, the regional CCD with different maladjusted levels is very close to the critical value of the previous level. It indicates that there is a lot of possibility for adjustment and improvement in the coordinated development of systems in various regions. Most regions are likely to reach the standard of primary coordination.
(3) In terms of spatial characteristics, there are obvious regional differences in the level of coupling coordination between the “LCD-TI-NU” system, showing “high in the central, low in the north and south,” “high in the east and low in the west” status. From the perspective of spatial correlation, the CCD has obvious clustering characteristics and positive spatial autocorrelation. The spatial correlation shows an increasing trend. Combined with the average CCD of each region from 2009 to 2019, most of the regions with higher values are concentrated in the eastern region. The lower values regions are concentrated in the western region. The central and eastern regions of the top 10 regions account for 80%. The second echelon is dominated by the central region. Most of the third echelon is the western regions.
(4) Among the obstacles affecting the LCD and the NU subsystems, the most prominent are economic indexes, mainly in the central and western regions. The eastern region is more subject to social security indexes. The spatial differences of obstacle indexes in the TI subsystem are not significant, and the obstacle indexes mainly reflect in the TI investment. In order to achieve system and regional coordination, it is necessary to strengthen cooperation among various departments, emphasize the integrity and synergy of policies and measures, focus on economic development, and then steadily promote social progress.

### Policy implications

NU advocates “people-oriented” and sustainable development. It has become a new driving force for China's economic and social development. Promoting LCD will inject green power into accelerating the construction of NU and urban-rural integration, thereby enhancing the vitality of sustainable development in the region. As a technical means and power guarantee, regional TI will also help to create a high-quality living space for living and working on the basis of improving the ability of LCD. Ultimately, the NU construction will be significantly improved. Therefore, promoting the coordinated development of LCD, TI and NU is an important way to promote the coordinated development of China's regions. It is also an important support for relevant policies and long-time outline plan. In view of the above conclusions, this paper puts forward the following suggestions:

(1) Use policy incentives to bridge the gap between NU and LCD. LCD and NU are both the result of a balance between economic development and policy guidance. This is also evident in the obstacle factors of the two subsystems. Firstly, with the growing demand of urban residents for high-quality public services, ecological environment and health and safety, the government needs to increase investment in public services, such as social security and employment expenditures, environmental protection expenditures, etc. The government should focus on regional infrastructure construction. It is necessary to increase the investment in regional greening and optimize the investment in public transportation. It is also important to provide facilities in infrastructure construction and to achieve equalization of basic public services in urban and rural areas. Furthermore, publicity and guidance should be done to promote low-carbon behaviors. Secondly, relevant departments can restrict the total carbon emissions of enterprises through environmental supervision policies. Whether to impose carbon tax to force high-carbon enterprises to reduce emissions from their own initiative needs to be seriously discussed in the future. The imposition of carbon tax will increase enterprises' cost, reduce enterprises' profit space, and indirectly reduce the number of enterprises employed. In the short term, it may lead to a reduction in labor market demand, slowing the flow of rural people to cities, and thus slowing down the speed of urbanization. It may also cause costs to be passed on to consumers, indirectly affect per capita income, and hinder the improvement of the NU level. Furthermore, the current mechanism is not perfect. Therefore, China currently mainly adopts the carbon emission trading mechanism as its own emission reduction tool. Thirdly, in order to optimize the relationship between the government and enterprises as soon as possible, government departments should speed up the construction of a green industry system and promote the transformation and upgrading of traditional industries to green industries. The market can spawn and expand a number of green, low-carbon enterprises and create a new job market. It is necessary to promote the integration of secondary and tertiary industries, add service links in the industrial chain, and further expand the demand for labor. While promoting green and low-carbon production, it will stimulate more employment opportunities and promote the development of new regional urbanization.
(2) Combined with geographical location and resource endowment, each region should address the imbalance of TI capabilities according to local conditions. Firstly, all regions should strengthen policies and capital investment in TI, and encourage universities and research institutes to carry out technological research. Institutions should increase financial support for excellent research projects, promote the transformation and implementation of project results, and then commend and reward front-line personnel who have made outstanding contributions. Secondly, each region needs to make effective use of local resources and location advantages. For example, the central and western regions can focus on creating competitive advantage such as low cost of living, livability and encouragement for entrepreneurship to attract innovative talents. The northeast region can highlight the dominant position of enterprises in innovation, and encourage enterprises to carry out technology introduction and innovation. Finally, expand radiation and spillover effects to narrow the development gap between regions. Due to the relatively slow development in the western region, the degree of talent agglomeration is low. The comprehensive development level and CCD in this area are lower than average. Therefore, some provinces can play the role of “leader”. For example, advantageous enterprises in Sichuan and Shaanxi can carry out cooperative investment in neighboring provinces, strengthen the regional cooperation, industrial connection and technological exchanges, and promote technological innovation and achievement transformation.
(3) LCD, TI, and NU should be integrated and developed to improve the coupling coordination level. The integration of TI and LCD will spawn a wave of green entrepreneurial teams, green enterprises and green industry activities. The integration of TI and NU will promote the construction of smart cities and the improvement of modernization levels. The integration of LCD and NU will help to build low-carbon cities. Therefore, some policies can exert force from these three aspects. Firstly, each region insists on taking green technology research as a breakthrough to incubate entrepreneurial teams with patented technologies. Furthermore, the government and the market cooperate to build a service platform for green innovation ecology, and then gather green technology-based enterprises. It is also necessary to encourage enterprises to innovate and promote breakthroughs in key green and low-carbon technologies. Secondly, in the process of NU construction, the government should increase TI investment and financial support to encourage renewable energy's utilization. Based on intelligence, relevant institutions can combine digital technology with traditional power electronic technology to promote the intelligence and informatization in urban infrastructure construction. Thirdly, strengthen policy support in fiscal, financial, planning and construction. Accelerate the construction of low-carbon cities, such as building a conservation-minded government, creating green communities, and promoting green buildings. It is also important to advocate the concept of low-carbon life, encourage citizens to use environment-friendly products, continue to promote garbage classification, and develop a circular economy.

### Contributions and limitations

The contributions of this paper mainly include the following aspects:

(1) The index system of LCD, TI, and NU are systematically constructed and supplemented. It can better evaluate and reflect the coordinated development characteristics, and make up for the lack of research on the relationship between the three systems in the existing literature. (2) The advantages of different research methods are combined to present a complete study. The analysis in terms of external representations of temporal evolution and spatial agglomeration strengthens the existing research on coupling coordination relationships, while complementary studies are conducted in internal mechanisms affecting the level of coupled coordination, expanding the scope of application of research methods across different fields and systems. (3) By means of the obstacle degree model, the obstacle indexes affecting the comprehensive and coordinated development level of each subsystem are analyzed. It would provide an effective decision-making basis for the formulation of the regional promotion. (4) In theory, it provides case data for regional governance theory. In practice, it is helpful for provinces and cities to implement targeted policy measures to solve such problems.

However, due to the availability of data and the limitation of research methods, this paper also has many limitations: (1) This paper only selected data at the provincial level, and yet the data at the city and county levels can better reflect regional differences. Future research can be supplemented and refined from the study area. (2) The “LCD-TI-NU” system involves multi-dimensional indexes, such as economy, society, environment, resources, and population. Future research can also supplement more indexes based on realistic background. For example, the proportion of green buildings and intelligent transportation can be supplemented in the index system of NU subsystem. (3) Subsequent research can use the spatial econometric model to investigate the influencing factors of the coupling coordination level, and decompose the influence effect. In this way, the direct and indirect effects of each factor can be explored more accurately from the temporal and spatial dimensions.

## Data availability statement

The original contributions presented in the study are included in the article/supplementary material, further inquiries can be directed to the corresponding author.

## Author contributions

YH contributions include writing original draft, investigation, and data curation. GL contributions include methodology, review, and editing. Both authors contributed to the article and approved the submitted version.

## Funding

This funding was supported by the Fundamental Research Funds for the Central Universities (Grant No. CCNU18ZYTS03).

## Conflict of interest

The authors declare that the research was conducted in the absence of any commercial or financial relationships that could be construed as a potential conflict of interest.

## Publisher's note

All claims expressed in this article are solely those of the authors and do not necessarily represent those of their affiliated organizations, or those of the publisher, the editors and the reviewers. Any product that may be evaluated in this article, or claim that may be made by its manufacturer, is not guaranteed or endorsed by the publisher.
